# Lifespan and Aggregate Size Variables in Specifications of Mortality or Survivorship

**DOI:** 10.1371/journal.pone.0084156

**Published:** 2014-01-15

**Authors:** Michael Epelbaum

**Affiliations:** Independent Multidisciplinary Scientist, Nashville, Tennessee, United States of America; Institut Pluridisciplinaire Hubert Curien, France

## Abstract

A specification of mortality or survivorship provides respective explicit details about mortality's or survivorship's relationships with one or more other variables (e.g., age, sex, etc.). Previous studies have discovered and analyzed diverse specifications of mortality or survivorship; these discoveries and analyses suggest that additional specifications of mortality or survivorship have yet to be discovered and analyzed. In consistency with previous research, multivariable limited powered polynomials regression analyses of mortality and survivorship of selected humans (Swedes, 1760–2008) and selected insects (caged medflies) show age-specific, historical-time-specific, environmental-context-specific, and sex-specific mortality and survivorship. These analyses also present discoveries of hitherto unknown lifespan-specific, contemporary-aggregate-size-specific, and lifespan-aggregate-size-specific mortality and survivorship. The results of this investigation and results of previous research help identify variables for inclusion in regression models of mortality or survivorship. Moreover, these results and results of previous research strengthen the suggestion that additional specifications of mortality or survivorship have yet to be discovered and analyzed, and they also suggest that specifications of mortality and survivorship indicate corresponding specifications of frailty and vitality. Furthermore, the present analyses reveal the usefulness of a multivariable limited powered polynomials regression model-building approach. This article shows that much has yet to be learned about specifications of mortality or survivorship of diverse kinds of individuals in diverse times and places.

## Introduction

A specification of mortality or survivorship provides explicit details about mortality's or survivorship's relationships with one or more other variables. For example, *X*-specific mortality or survivorship provides explicit details about mortality's or survivorship's relationship with variable *X*. Previous investigations present discoveries and analyses of diverse specifications of mortality or survivorship, as illustrated by discoveries and analyses of age-specific [1]–[40], environmental-context-specific [10], [13], [17], [35], [41], [42], historical-time-specific [17], [43]–[47], physical-size-specific [2], [10], [12], [20], [34], [35], [48]–[53], sex-specific [10], [35], [54], birth-cohort-specific [23], [36]–[40], [44], [47], [55], [56], exposure-specific [12], [13], [44], [47], [57], density-specific [10], [17], [41], [58]–[60], and disease-specific [61] mortality or survivorship. These considerations suggest that additional specifications of mortality or survivorship have yet to be discovered and analyzed. This article presents discoveries and analyses of hitherto unknown lifespan-specific, contemporary-aggregate-size-specific, and lifespan-aggregate-size-specific mortality and survivorship.

Lifespan is the total time span of an individual's existence [21], [62], such that *L_iq_ = L_i_ = t_iz_ – t_i0_*, where *L_iq_* refers to the lifespan of a natural or artificial individual *i* at time *t_q_*, *z≥q*, *t_iz_* is the time of the individual's cessation of existence, *t_i0_* is the time of the individual's initiation of existence, and *L_i_* is constant for all *t_q_* in *t_i0_:t_iz_*. The time of birth typically indicates time *t_i0_*, and the time of death typically indicates time *t_iz_*, but these typical notions of time of birth and time of death as limits of lifespan do not apply to all kinds of individuals [10], [20], [63]. The lifespan aggregate includes all the individuals that are identically characterized with respect to lifespan and every other condition in a data set. The individuals that are included in a lifespan aggregate begin their existence in coexistence at the beginning of the lifespan, they coexist through said lifespan, and they cease to exist and cease to coexist at the conclusion of this lifespan. Therefore, a lifespan aggregate's composition and size are constant from the time of the initiation of existence of this aggregate to the time of its cessation of existence. In some cases, the lifespan aggregate consists only of a respective single natural or artificial individual, but in many cases the lifespan aggregate consists of more than one individual. An individual's lifespan aggregate is included in every contemporary aggregate of this individual. The contemporary aggregate includes all the individuals that are identically characterized with respect to every condition in a data set at a point of cessation or continuation of existence, except that these individuals share or do not share an identical lifespan. These considerations indicate that the contemporary aggregate's composition and size are time-specific and changeable through time. Additionally, the size of an individual's contemporary aggregate is equal to – or greater than – the size of this individual's corresponding lifespan aggregate.

Every natural or artificial individual is characterized by a lifespan, a contemporary aggregate, and a lifespan aggregate at every point of continuation of existence (i.e., survivorship) and at the point of cessation of existence (i.e., mortality). Previous investigations posit that age-specific aggregates are characterized by a “longevity factor” [5]–[7]; this longevity factor has been implemented in logistic models of mortality or survivorship [3], [5]–[7] and in frailty models of survival time [15], [64]. However, lifespan-specific, contemporary-aggregate-size-specific, and lifespan-aggregate-size-specific mortality or survivorship have not been discovered or analyzed in previous empirical research. Therefore, it is useful to search for lifespan-specific, contemporary-aggregate-size-specific, and lifespan-aggregate-size-specific mortality or survivorship. This search is conducted here in empirical analyses of mortality and survivorship of selected humans and selected insects.

## Materials and Methods

### Data

Deaths 1×1 and exposures 1×1 tables (last modified on 14 July, 2010) from the Human Mortalities Database are employed here in the compilation of data on aggregate age-sex-year-specific deaths and age-sex-year-specific exposures of males and females in ages 0 to 110+ in Sweden 1751–2008 [65]. Computer intensive analyses impose restrictions on the size of the data file for the present analyses. Therefore, the analytic data file is restricted here to 188,087 weighted cases with 79,164,608 events of death or survival of all individuals born in Sweden in decennial years 1760–1930, with deaths occurring between 1760 and 2008. The selected aggregate data are converted here to yearly events of each individual's death or survival, where each individual-level case is weighted by its corresponding number of age-lifespan-sex-specific identical individuals (i.e., the number of sex-specific individuals who are born in the year of birth of the criterion individual and who die in the year of death of the criterion individual). Each case includes data on an age-sex-year-specific event of death or survival of one individual, year of the event, the individual's sex, the individual's age at the time of the event, the individual's lifespan, number of age-lifespan-sex-specific identical individuals (i.e., this is the weight variable in the analyses, and it is also the lifespan aggregate size variable in the respective models of mortality and survivorship), and the number of age-sex-specific individuals that are exposed to the risk of death and prospect of survival during the year of the event (i.e., this is the contemporary aggregate size variable in the respective models of the selected humans' mortality and survivorship).

The data on mortality and survivorship of the selected insects – Mediterranean fruit flies, *Ceratitis capitata*, commonly known as medflies – were collected in 1991 at the Moscamed medflies mass-rearing facility in Metapa, a small village located about 20 kilometers from the city of Tapachula in the state of Mexico [9], [10]. These data have been previously analyzed – using diverse compilations and methods – in studies that have been reported in diverse publications [9], [10], [27]–[29]. The original data file contains information on numbers of age-cage-and-sex-specific deaths of 1,203,646 male and female medflies, where insects are distributed in 167 cages, and where the numbers of age-cage-sex-specific dead individuals are counted daily [66]. Computer intensive analyses impose restrictions on the size of the data file that is analyzed here. Therefore, the analytic data file is restricted here to cases of physical size #5 and birth aggregate batch #2. In these selected cases, individuals lived and died in one of thirteen cages, where the cages averaged 3,646.3 sex-specific insects per cage at age 0 to 1 days. These aggregate data are converted here to daily events of each individual's death or survival, where each case is weighted by the number of sex-cage-specific individuals that were born in the day of birth of the criterion individual and that died in the day of death of the criterion individual. The resultant analytic data file includes 50,716 cases with 2,211,782 events of individual insects' deaths or survivals. Each case includes data on an age-cage-and-sex-specific event of death or survival of one individual, the individual's sex, the individual's age at the time of the event, the individual's lifespan, cage specifier, number of corresponding age-lifespan-cage-sex-specific identical individuals (i.e., number of cage-sex-specific individuals with identical birth day and identical death day to the criterion individual, which is the weight variable in the analyses, and which is also the lifespan aggregate size variable in the respective models of mortality and survivorship), and the number of age-cage-sex-specific individuals that are exposed to the risk of death and prospect of survival during the day of the event (i.e., this variable is also the contemporary aggregate size variable in the respective models of the selected insects' mortality and survivorship).

### Model-building approach

Mortality refers here to cessation of existence of an individual, and survivorship refers here to continuation of existence of an individual. Therefore, an explanatory model of mortality or survivorship – i.e., a model that is dedicated to the explanation of an individual's cessation or continuation of existence – requires a binary response model. Additionally, multiple specifications of mortality or survivorship – and avoidance of the omitted variables bias in models of mortality or survivorship [67]–[71] – require multivariable models. Furthermore, previous research shows that trajectories of specific mortality or survivorship tend to be nonlinear [31], [32]; therefore, the explanatory multivariable binary response model of mortality or survivorship should allow for nonlinearity. Previous research also shows that mortality and survivorship correspond to power laws and scaling laws [22], [26], [30], [35], [48]–[53], [60], [72]–[81]; therefore, the explanatory multivariable nonlinear binary response model of mortality or survivorship should enable investigation of power laws and scaling laws. The multivariable fractional polynomials regression model-building approach [31], [32], [34] enables investigation of explanatory multivariable nonlinear binary response models of mortality or survivorship. However, by allowing more than one power coefficient for each relevant right-hand side variable and by not searching for precise power coefficients, this model-building approach may disable the investigation of power laws and scaling laws of mortality or survivorship. Related to the multivariable fractional polynomials regression model-building approach, a multivariable limited powered polynomials regression model-building approach enables investigation of explanatory multivariable nonlinear binary response models, power laws, and scaling laws.

A multivariable limited powered polynomials regression model is specified here with
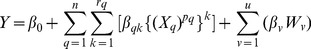
(1)where – in the present context – the left-hand side variable *Y* denotes mortality *M* or survivorship *S*, *β* denotes a regression coefficient, *X* denotes an ordinal or higher-level variable, and *W* denotes a categoric variable. In this regression model, a distinct precise power coefficient *p_q_* of a distinct variable *X_q_* is common to all *k* in each limited power series 
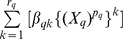
, and length *r_q_* of each of these limited power series is distinct to each variable *X_q_*. These characteristics of the multivariable limited powered polynomials regression model enable investigation of power laws, scaling laws, and post-estimation marginal probabilities and derivatives for each 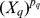 variable. In the following example of a multivariable limited powered polynomials regression model
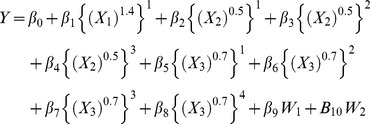
(2)variable *X_1_* has a *q = 1* index, a power coefficient *p_1_ = 1.4*, a limited power series of length *r_1_ = 1*, and one respective regression coefficient *β*; variable *X_2_* has a *q = 2* index, a power coefficient *p_2_ = 0.5*, a limited power series of length *r_2_ = 3*, and three respective regression coefficients *β*; variable *X_3_* has a *q = 3* index, a power coefficient *p_3_ = 0.7*, a limited power series of length *r_3_ = 4*, and four respective regression coefficients *β*; categoric variable *W_1_* has a *v = 1* index and one respective regression coefficient *β*; and categoric variable *W_2_* has a *v = 2* index and one respective regression coefficient *β*. The multivariable limited powered polynomials regression model in this example includes eleven regression coefficients *β* that are distributed as follows: one coefficient *β* for the intercept, eight coefficients *β* for the three *X_q_* variables and their respective limited power series, and two regression coefficients *β* for the two respective *W* variables. The model in this example enables investigation of power laws, scaling laws, and respective post-estimation marginal probabilities and derivatives for 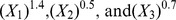.

### Statistical analyses

Analyses of mortality and survivorship of the selected humans analyze the following multivariable limited powered polynomials binary random effects weighted model:
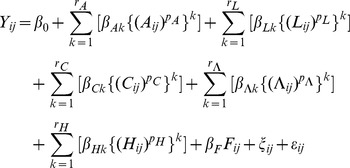
(3)where *Y_ij_* refers to mortality *M_ij_* or survivorship *S_ij_* of an individual human *i* that continues to exist (i.e., *M_ij_* = 0 and *S_ij_* = 1) or ceases to exist (i.e., *M_ij_* = 1 and *S_ij_* = 0) at observation *j*; *A_ij_*, *L_ij_*, *C_ij_*, *Λ_ij_*, *F_ij_*, and *H_ij_* are respective right-hand side variables corresponding to individual *i* at observation *j*; *A* denotes age, *L* denotes lifespan, *C* denotes contemporary aggregate size, *Λ* (the Greek capital letter *Lambda*) denotes lifespan aggregate size, *H* denotes historical time, and *F* (in reference to being or not being female) denotes sex; *ξ_ij_* denotes a random effects component corresponding to individual *i* at observation *j*; and *ε_ij_* denotes an error corresponding to individual *i* at observation *j*. Every 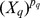 in Model (3) is a power transformation of a corresponding variable *X_q_* using a corresponding specific power coefficient *p_q_* (e.g., 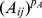 is a power transformation of *A_ij_*). Previous research provides evidence of unobserved heterogeneity in models of mortality or survivorship [9], [13]–[16], [28]; by denoting a random effects component of individual *i* at observation *j*, coefficient *ξ_ij_* in Model (3) accommodates and implements unobserved heterogeneity [82]. Additionally, previous research shows that regression models of mortality, survivorship, and other phenomena are often encumbered by the age-period-cohort problem (also known as the “APC conundrum”) of separating the effects of age-groups, periods, and cohorts in regression models [23], [36]–[40]. Inclusion of the variables age, lifespan, contemporary aggregate size, lifespan aggregate size, and historical time variables as separate and distinct variables in Model (3) shows that this model is not encumbered by the age-period-cohort problem.

Corresponding analyses of mortality and survivorship of the selected insects analyze the following multivariable limited powered polynomials binary random effects weighted model:
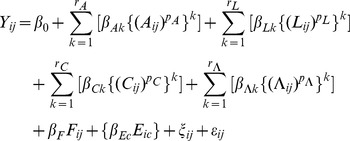
(4)where *Y_ij_*, *A_ij_*, *L_ij_*, *C_ij_*, *Λ_ij_*, *F_ij_*, *p_q_*, *k*, *β_qk_*, *ξ_ij_*, and *ε_ij_* denote as in Model (3); *E_ic_* denotes the environmental context *E* of individual *i*, such that *c* in *β_Ec_* and *E_ic_* denotes a specific cage *c*, such that *c* = 1∶13 cages, such that Model (4) includes one of 13 respective terms *{β_Ec_E_ic_}*, such that one of these 13 respective terms applies to a respective individual *i*.

Statistical analyses of limited powered polynomials binary random effects weighted regression Models (3) and (4) are conducted here using the Stata software [83]. Stata restricts the statistical analyses of random effects binary response models to respective analyses of logit, probit, and complementary log-log models with a Gaussian distribution of unobserved heterogeneity. Goodness-of-fit (GOF) of a model is indicated here by minimization of the Akaike information criterion, AIC, and minimization of the Bayesian information criterion, BIC [83]–[85]. Statistical analyses of Models (3) and (4) consist here of data-driven stepwise tests of improvements in GOF in respective weighted random effects logit, probit, or complementary log-log regression analyses of these models.

Initial steps in the stepwise analyses employ *k = 1* of all *n* right-hand side variables 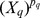 of Model (3) or (4), testing diverse power coefficients *p_q_* (using *ln(X_q_)* for *p_q_ = 0*), searching for the power coefficient *p_q_* for each specific 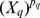 variable that most improves the model's GOF, stopping respective testing of a specific 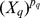 when a specific change in *p_q_* for this specific 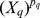 ceases to improve the model's GOF, dropping variables 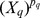 that fail to improve the model's GOF, and retaining variables 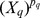 that most improve the model's GOF. The distinct power coefficients *p_q_* of respective distinct variables 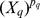 that are retained when *k = 1* are kept constant in all the subsequent GOF tests of 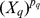 variables with *k>1*. If GOF tests of 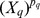 variables with *k>1* improve the model's GOF, then increasing *k* and continuing stepwise reiterations of these tests, until no further improvements in the model's GOF are achieved. The best-fitting model is also required to enable calculations of post-estimation marginal probabilities and marginal derivatives; if these calculations are not achieved then calculations are attempted with the most preceding improved model until success in such calculations is achieved. Thus, a best-fitting model here is the model whose right-hand side variables *X_q_* and *W*, power coefficients *p_q_*, and respective limited power series coefficients *k* and *r_q_* minimize AIC and BIC and enable successful calculations of post-estimation marginal probabilities and marginal derivatives. Statistical analyses culminate here in selections of a best-fitting model of the selected humans' mortality, a best-fitting model of the selected humans' survivorship, a best-fitting model of the selected insects' mortality, and a best-fitting model of the selected insects' survivorship. The best-fitting models yield *z*-ratios and respective probabilities *P(|z|)* for these ratios, where *z = β/SE(β)*. Coefficients *P(|z|)* serve here as respective indicators of respective specifications of mortality or survivorship.

## Results

### Best-fitting models and specifications of mortality and survivorship

The analyses yield a best-fitting multivariable limited powered polynomials random effects logit weighted model of the selected humans' mortality. Table 1 presents respective *β*, *p_q_*, *k*, *SE(β)*, *z*, and *P(|z|*) coefficients of this best-fitting model of the selected humans' mortality. This model is computed on the basis of Model (3) and – employing *β* coefficients from Table 1 – it is specified with
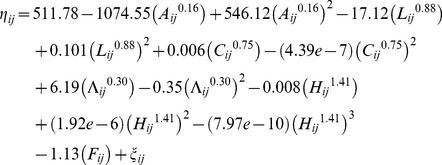
(5)


**Table 1:** Coefficients of the best-fitting multivariable limited powered polynomials random effects weighted logit model of the selected humans' mortality.^1^

Variable	*β* Index	*β*	*SE(β)*	*z-ratio*	*P*(|*z*|)	*β's* 95% Confidence Interval	
*Constant*	*β_0_*	511.7836	0.577069	886.87	0.00	510.6526	512.9146
*A^0.16^*	*β_A1_*	−1074.55	1.208563	−889.12	0.00	−1076.92	−1072.19
*(A^0.16^)^2^*	*β_A2_*	546.1184	0.613552	890.09	0.00	544.9158	547.3209
*L^0.88^*	*β_L1_*	−17.1193	0.019416	−881.73	0.00	−17.1574	−17.0813
*(L^0.88^)^2^*	*β_L2_*	0.100631	0.00012	839.59	0.00	0.100396	0.100866
*C^0.75^*	*β_C1_*	0.006233	2.08e-05	299.18	0.00	0.006192	0.006273
*(C^0.75^)^2^*	*β_C2_*	−4.39e-07	3.42e-09	−128.68	0.00	−4.46e-07	−4.33e-07
*Λ^0.3^*	*β_Λ1_*	6.186891	0.00919	673.25	0.00	6.16888	6.204902
*(Λ^0.3^)^2^*	*β_Λ2_*	−0.34869	0.000512	−681.26	0.00	−0.34969	−0.34768
*F*	*β_F_*	−1.12889	0.004455	−253.37	0.00	−1.13762	−1.12016
*H^1.41^*	*β_H1_*	−0.00784	3.58e-05	−219.24	0.00	−0.00791	−0.00777
*(H^1.41^)^2^*	*β_H2_*	1.92e-06	2.90e-08	66.06	0.00	1.86e-06	1.97e-06
*(H^1.41^)^3^*	*β_H3_*	−7.97e-10	7.70e-12	−103.52	0.00	−8.12e-10	−7.82e-10

^1^ Variables are right-hand side (rhs) variables of the best-fitting model. Variables include: *A* denoting age (in years), *L* denoting lifespan (in years), *C* denoting contemporary aggregate size, *Λ* denoting lifespan aggregate size, *F* denoting sex, and *H* denoting historical time (i.e., indicated by a specific year). Coefficient *β* denotes a regression coefficient of the respective best-fitting model, *SE(β)* denotes the standard error of *β*, *z* denotes a specific *z*-ratio calculated with *z = β/SE(β)*, and *P(|z*|) denotes a respective probability of *|z|*.

such that *M_ij_* = *exp(η_ij_)/{1+ exp(η_ij_)}*, where *i* denotes an individual, *j* is the consecutive number for the respective consecutive observation of this individual's cessation or continuation of existence, *M_ij_* denotes the logit fitted probability of mortality of individual *i* at observation *j*, *A_ij_* denotes the individual's age (in years) at observation *j*, *L_ij_* denotes the individual's lifespan (in years) at observation *j*, *C_ij_* denotes the individual's contemporary aggregate size at observation *j*, *Λ_ij_* denotes the individual's lifespan aggregate size at observation *j*, *H_ij_* denotes the individual's historical time at observation *j* where *j* denotes a calendar year transformed to a sequential number, *F_ij_* = 1 when the individual is female and *F_ij_* = 0 otherwise, and *ξ_ij_* denotes the random effects component corresponding to individual *i* at observation *j*.

The analyses also yield a corresponding best-fitting multivariable limited powered polynomials random effects logit weighted model of the selected humans' survivorship. Table 2 presents respective *β*, *p_q_*, *k*, *SE(β)*, *z*, and *P(|z|*) coefficients of this best-fitting model of the selected humans' survivorship. This model is computed on the basis of Model (3) and – employing *β* coefficients from Table 2 – it is specified with
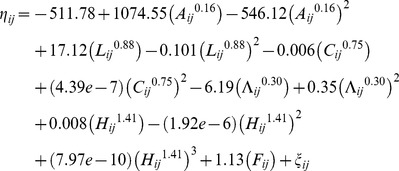
(6)


**Table 2:** Coefficients of the best-fitting multivariable limited powered polynomials random effects weighted logit model of the selected humans' survivorship.^1^

Variable	*β* Index	*β*	*SE(β)*	*z-ratio*	*P*(|*z*|)	*β's* 95% Confidence Interval	
*Constant*	*β_0_*	−511.784	0.577069	−886.87	0.00	−512.915	−510.653
*A^0.16^*	*β_A1_*	1074.553	1.208563	889.12	0.00	1072.185	1076.922
*(A^0.16^)^2^*	*β_A2_*	−546.118	0.613552	−890.09	0.00	−547.321	−544.916
*L^0.88^*	*β_L1_*	17.11934	0.019416	881.73	0.00	17.08128	17.15739
*(L^0.88^)^2^*	*β_L2_*	−0.10063	0.00012	−839.59	0.00	−0.10087	−0.1004
*C^0.75^*	*β_C1_*	−0.00623	2.08e-05	−299.18	0.00	−0.00627	−0.00619
*(C^0.75^)^2^*	*β_C2_*	4.39e-07	3.42e-09	128.68	0.00	4.33e-07	4.46e-07
*Λ^0.3^*	*β_Λ1_*	−6.18689	0.00919	−673.25	0.00	−6.2049	−6.16888
*(Λ^0.3^)^2^*	*β_Λ2_*	0.348686	0.000512	681.26	0.00	0.347683	0.349689
*F*	*β_F_*	1.128888	0.004455	253.37	0.00	1.120156	1.137621
*H^1.41^*	*β_H1_*	0.007839	3.58e-05	219.24	0.00	0.007769	0.007909
*(H^1.41^)^2^*	*β_H2_*	−1.92e-06	2.90e-08	−66.06	0.00	−1.97e-06	−1.86e-06
*(H^1.41^)^3^*	*β_H3_*	7.97e-10	7.70e-12	103.52	0.00	7.82e-10	8.12e-10

^1^ As in the footnote of Table 1.

such that *S_ij_* = *exp(η_ij_)/{1+ exp(η_ij_)}*, where *S_ij_* denotes the logit fitted probability of survival of individual *i* at observation *j*, and all other denotations are as in Model (5).

The analyses yield a best-fitting multivariable limited powered polynomials random effects logit weighted model of the selected insects' mortality. Table 3 presents respective *β*, *p_q_*, *k*, *SE(β)*, *z*, and *P(|z|*) coefficients of this best-fitting model of the selected insects' mortality. This model is computed on the basis of Model (4) and – employing *β* coefficients from Table 3 – it is specified with
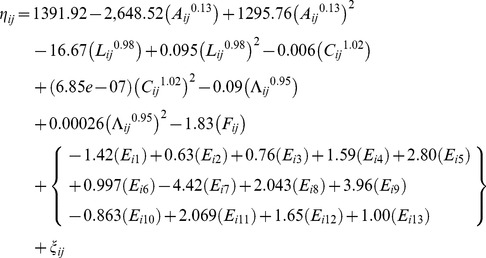
(7)


**Table 3:** Coefficients of the best-fitting multivariable limited powered polynomials random effects weighted logit model of the selected insects' mortality.^1^

Variable	*β* Index	*β*	*SE(β)*	*z-ratio*	*P*(|*z*|)	*β's* 95% Confidence Interval	
*Constant*	*β_0_*	1391.92	8.754245	159.00	0.00	1374.76	1409.08
*A^0.13^*	*β_A1_*	−2648.52	16.6719	−158.86	0.00	−2681.2	−2615.85
*(A^0.13^)^2^*	*β_A2_*	1295.76	8.161646	158.76	0.00	1279.76	1311.76
*L^0.98^*	*β_L1_*	−16.67	0.106237	−156.94	0.00	−16.88	−16.46
*(L^0.98^)^2^*	*β_L2_*	0.095159	0.00062	153.51	0.00	0.093944	0.096374
*C^1.02^*	*β_C1_*	−0.00632	0.000103	−61.38	0.00	−0.00652	−0.00612
*(C^1.02^)^2^*	*β_C2_*	6.85e-07	1.55e-08	44.10	0.00	6.54e-07	7.15e-07
*Λ^0.95^*	*β_Λ1_*	−0.09025	0.001541	−58.58	0.00	−0.09327	−0.08723
*(Λ^0.95^)^2^*	*β_Λ2_*	0.000263	4.91e-06	53.64	0.00	0.000254	0.000273
*F*	*β_F_*	−1.82694	0.040958	−44.61	0.00	−1.90722	−1.74667
*E_1_*	*β_E1_*	−1.41705	0.096002	−14.76	0.00	−1.60521	−1.22889
*E_2_*	*β_E2_*	0.631117	0.102214	6.17	0.00	0.430781	0.831452
*E_3_*	*β_E3_*	0.756026	0.099505	7.60	0.00	0.561	0.951052
*E_4_*	*β_E4_*	1.597167	0.09196	17.37	0.00	1.416928	1.777405
*E_5_*	*β_E5_*	2.7985	0.096029	29.14	0.00	2.610285	2.986714
*E_6_*	*β_E6_*	0.996794	0.088613	11.25	0.00	0.823116	1.170472
*E_7_*	*β_E7_*	−4.41742	0.104782	−42.16	0.00	−4.62279	−4.21205
*E_8_*	*β_E8_*	2.042812	0.100085	20.41	0.00	1.846648	2.238976
*E_9_*	*β_E9_*	3.962415	0.093918	42.19	0.00	3.77834	4.146491
*E_10_*	*β_E10_*	−0.86276	0.10499	−8.22	0.00	−1.06854	−0.65699
*E_11_*	*β_E11_*	2.069779	0.097441	21.24	0.00	1.878799	2.26076
*E_12_*	*β_E12_*	1.655298	0.08756	18.9	0.00	1.483683	1.826912

^1^ Variables are right-hand side variables of the best-fitting model. Variables' respective designators include: *A* designating age (in days), *L* designating lifespan (in days), and *E* designating environmental context (i.e., indicated by a specific cage index). All else is as in the footnote of Table 1.

where *M_ij_*, *L_ij_*, *C_ij_*, *Λ_ij_*, *F_ij_*, and *ξ_ij_* denote as in Model (5), *A_ij_* denotes the age (in days) of individual *i* at observation *j*, and coefficients *E_ic_* respectively denote the environmental context of an individual *i* in one of *c = 1∶13* cages, such that only one of the 13 terms of coefficients *E_ic_* applies to individual *i* within parentheses *{}* of Model (7).

The analyses also yield a best-fitting multivariable limited powered polynomials random effects complementary log-log weighted model of the selected insects' survivorship. Table 4 presents respective *β*, *p_q_*, *k*, *SE(β)*, *z*, and *P(|z|*) coefficients of this best-fitting model of the selected insects' survivorship. This model is computed on the basis of Model (4) and – employing *β* coefficients from Table 4 – it is specified with
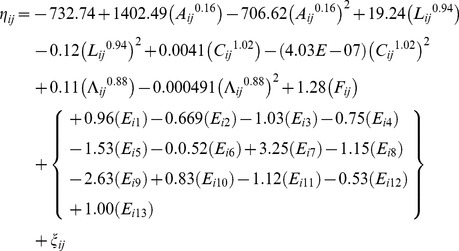
(8)


**Table 4:** Coefficients of the best-fitting multivariable limited powered polynomials random effects weighted complementary log-log model of the selected insects' survivorship.^1^

Variable	*β* Index	*β*	*SE(β)*	*z-ratio*	*P*(|*z*|)	*β's* 95% Confidence Interval	
*Constant*	*β_0_*	−732.741	7.57595	−96.72	0.00	−747.59	−717.893
*A^0.16^*	*β_A1_*	1402.486	14.67483	95.57	0.00	1373.72	1431.25
*(A^0.16^)^2^*	*β_A2_*	−706.621	7.484498	−94.41	0.00	−721.29	−691.951
*L^0.94^*	*β_L1_*	19.23612	0.212374	90.58	0.00	18.81988	19.65237
*(L^0.94^)^2^*	*β_L2_*	−0.11759	0.001355	−86.8	0.00	−0.12025	−0.11493
*C^1.02^*	*β_C1_*	0.004073	0.000101	40.4	0.00	0.003875	0.004271
*(C^1.02^)^2^*	*β_C2_*	−4.03e-07	1.49e-08	−26.99	0.00	−4.32e-07	−3.74e-07
*Λ^0.88^*	*β_Λ1_*	0.112845	0.002865	39.38	0.00	0.10723	0.118461
*(Λ^0.88^)^2^*	*β_Λ2_*	−0.00049	1.38e-05	−35.57	0.00	−0.00052	−0.00046
*F*	*β_F_*	1.282721	0.041707	30.76	0.00	1.200977	1.364465
*E_1_*	*β_E1_*	0.964724	0.10298	9.37	0.00	0.762886	1.166561
*E_2_*	*β_E2_*	−0.66944	0.121445	−5.51	0.00	−0.90747	−0.43141
*E_3_*	*β_E3_*	−1.03296	0.102814	−10.05	0.00	−1.23448	−0.83145
*E_4_*	*β_E4_*	−0.74903	0.115518	−6.48	0.00	−0.97544	−0.52262
*E_5_*	*β_E5_*	−1.53755	0.118571	−12.97	0.00	−1.76995	−1.30516
*E_6_*	*β_E6_*	−0.52188	0.106935	−4.88	0.00	−0.73147	−0.31229
*E_7_*	*β_E7_*	3.253259	0.103586	31.41	0.00	3.050235	3.456284
*E_8_*	*β_E8_*	−1.1545	0.141288	−8.17	0.00	−1.43142	−0.87758
*E_9_*	*β_E9_*	−2.6341	0.115133	−22.88	0.00	−2.85975	−2.40844
*E_10_*	*β_E10_*	0.831803	0.116184	7.16	0.00	0.604087	1.059518
*E_11_*	*β_E11_*	−1.1248	0.131534	−8.55	0.00	−1.3826	−0.867
*E_12_*	*β_E12_*	−0.53327	0.109138	−4.89	0.00	−0.74718	−0.31936

^1^ As in the footnote of Table 3.

such that *S_ij_* = *1 - exp{-exp(η_ij_)}*, where *S_ij_* denotes the complementary log-log fitted probability of survival of individual *i* at observation *j*, and all other denotations are as in Model (7).

Coefficients *P(|z|*) in Tables 1 and 3 provide evidence of respective age-specific, lifespan-specific, contemporary-aggregate-size-specific, historical-time-specific, environmental-context-specific, and sex-specific mortality. Similarly, coefficients *P(|z|*) in Tables 2 and 4 provide evidence of respective age-specific, lifespan-specific, contemporary-aggregate-size-specific, historical-time-specific, environmental-context-specific, and sex-specific survivorship. Thus, as noted, the best-fitting models yield respective evidence of respective age-specific, lifespan-specific, contemporary-aggregate-size-specific, lifespan-aggregate-size-specific, historical-time-specific, environmental-context-specific, and sex-specific mortality and survivorship of the selected humans and the selected insects in this investigation.

## Discussion

Making reference to existence of any natural or artificial individual (i.e., individuals from apples to zithers; for example, particles, plants, planets, viruses, insects, humans, bicycles, books, and poems), survivorship refers here to continuation of existence, and mortality refers here to cessation of existence. Every natural or artificial individual is characterized by age, lifespan, contemporary aggregate size, lifespan aggregate size, historical time, and environmental context at every point of continuation of existence and at the point of cessation of existence. Similarly, every sexual individual is characterized by sex at every point of continuation of existence and at the point of cessation of existence. These considerations – and the specifications in the present investigation and in past research – provide guidance to the inclusion of the variables age, lifespan, contemporary aggregate size, lifespan aggregate size, historical time, and environmental context in regression models of mortality or survivorship of diverse kinds of natural or artificial individuals in diverse times and places. Similarly, these considerations – and the specifications in the present investigation and in past research – provide guidance to the inclusion of the variable sex in regression models of mortality or survivorship of diverse kinds of sexual individuals in diverse times and places.

As noted, respective age-specific, historical-time-specific, environmental-context-specific, and sex-specific mortality or survivorship have already been discovered and analyzed in previous empirical research. However, respective lifespan-specific, contemporary-aggregate-size-specific, and lifespan-aggregate-size-specific mortality or survivorship have not been discovered or analyzed in previous empirical research. The present discoveries and analyses of hitherto unknown lifespan-specific, contemporary-aggregate-size-specific, and lifespan-aggregate-size-specific mortality and survivorship reveal that much has yet to be learned about these specifications among diverse kinds of individuals in diverse times and places. Additionally, the new discoveries and analyses strengthen the suggestion that additional specifications of mortality or survivorship have yet to be discovered and analyzed.

As noted, previous research shows that regression models of mortality or survivorship are encumbered by the omitted variables bias [67]–[71] and by unobserved heterogeneity bias [9], [13]–[16], [28]. The present investigation suggests that addition of lifespan, contemporary aggregate size, or lifespan aggregate size variables to the right-hand side of regression models of mortality or survivorship – and employment of multivariable limited powered polynomials regression models – reduce or eliminate these biases. Furthermore, as noted, previous research shows that trajectories of mortality or survivorship tend to be nonlinear [31], [32], that mortality and survivorship correspond to power laws and scaling laws [22], [26], [30], [35], [48]–[53], [60], [72]–[81], and that previous models of mortality or survivorship are encumbered by the age-period-cohort problem of separating the effects of age-groups, periods, and cohorts in regression models [23], [36]–[40]. The present investigation suggests that multivariable limited powered polynomials binary regression models of mortality or survivorship help capture nonlinearity, contribute to analyses of power laws and scaling laws, and provide a useful solution to the age-period-cohort problem. These suggestions can now be investigated further.

This investigation and previous research elucidate – and are elucidated by – considerations of frailty and vitality. Frailty is typically conceptualized in negative terms conveying vulnerability, susceptibility, weakness, debility, defenselessness, helplessness, exposure, liability, lack, absence, decay, decline, exhaustion, or depletion. Vitality is typically conceptualized in positive terms conveying liveliness, vigor, strength, resistance, robustness, animation, verve, dynamism, vim, resistance, success, accomplishment, achievement, or expansion. Notions of frailty and vitality have been prevalent in many cultures throughout human evolution and history, as exemplified by expressions of these notions in vitalism, *yinyang*, *élan vital*, or *conatus*. There is ample research on frailty in mortality or survivorship [14]–[16], [19], [64], [86]–[91], and there is also ample research on vitality in mortality or survivorship [1], [2], [11], [12], [19], [22], [28], [92]–[115]. Previous research posits the existence of an “inherent vitality” that is defined as “the total potential capacity of an [individual] to perform vital actions, in the complete absence of matter or energy of exogenous derivation” ([2], pp. 108, 147). This conception of inherent vitality – together with general notions of frailty and vitality as well as previous research on frailty and vitality in mortality and survivorship – suggest that each natural or artificial individual is characterized by lifespan-specific vitality and a corresponding lifespan-specific frailty. Additionally, previous research focuses on attritions of individuals from respective frailty-based aggregates – and retentions of individuals in respective vitality-based aggregates – through the life course [9], [10], [13]–[16], [28], [64], [89], [90], [100], [116]–[127], suggesting an affinity between frailty and aggregate-size-specific mortality and suggesting an affinity between vitality and aggregate-size-specific survivorship. These suggestions are generalized here by positing that specifications of mortality indicate corresponding specifications of frailty and by positing that specifications of survivorship indicate corresponding specifications of vitality. These suggested generalizations reveal that much remains to be learned about specifications of mortality, survivorship, frailty, and vitality of diverse kinds of individuals in diverse times and places.
